# The Molecular Mechanism of FABP4 Inhibition Effects of GAS and 4-HBA in *Gastrodia elata* Blume Was Discussed Based on NMR and Molecular Docking

**DOI:** 10.1155/2024/6599029

**Published:** 2024-05-08

**Authors:** Yuyu Yang, Shihan Liu, Wenfang Jin, Zengyi Qu, Baolei Fan

**Affiliations:** ^1^Hubei University of Science and Technology, No. 88, Xianning Avenue, Xianan District, Xianning 437000, China; ^2^Hubei Provincial Key Laboratory of Radiation Chemistry and Functional Materials, Hubei University of Science and Technology, No.88, Xianning Avenue, Xianan District, Xianning 437000, China

## Abstract

To isolate gastrodin (GAS), 4-hydroxybenzyl alcohol (4-HBA), and phenolic compounds from Chinese medicine *Gastrodia elata* Blume, and to explore the binding mode of fatty acid binding protein 4 (FABP4/aP2) that is closely related to macrophage inflammation, we study their anti-inflammatory targets. After the ultrasonic extraction of the main active components with 70% ethanol, three resins and three eluents were selected, and eight phenolic monomers with similar structures, such as gastrodin and 4-hydroxybenzyl alcohol, were isolated from *Gastrodia elata* by AB-8 macroporous resin and silica gel column chromatography and eluted with the CHCl_3_-MeOH gradient. Their structures were identified by HPLC and nuclear magnetic resonance (NMR). The FABP4 protein was added to GAS and 4-HBA, and the NMR experiment was performed to observe ligand binding. Finally, according to the spectral information of STD-NMR and molecular docking technology, the interaction between ligands and protein was studied. The fluorescence competition experiment confirmed that both GAS and 4-HBA were in the binding cavity of FABP4. Moreover, 3-phenoxy-2-phenylbenzoic acid (PPA) is a possible inhibitor of FABP4, reducing macrophage-related inflammation and endoplasmic reticulum stress. This work provides a new basis for the anti-inflammatory mechanism of *Gastrodia elata*, paving the way for the research and development of FABP4 inhibitor drugs.

## 1. Introduction

Adipocyte fatty-acid-binding protein (FABP4) is expressed in adipocytes and macrophages and integrates inflammatory and metabolic responses [1]. Studies in FABP4-deficient mice have shown that this lipid chaperone has a significant role in several aspects of metabolic syndrome, including type 2 diabetes and atherosclerosis [1]. Foam cells on the inner membrane of the aorta in the atherosclerosis mice were significantly reduced in the presence of the FABP4 inhibitor (BMS309403, 25 mM). The expression of these cytokines (MCP-1, interleukin (IL)1*β*, IL6, and tumor necrosis factor (TNF) in macrophages) was significantly reduced in the FABP4-expressing macrophages treated with the FABP4 inhibitor [1]. On the other hand, gastrodin attenuates the foam cells on the inner membrane of the inner membrane of aorta in atherosclerosis mice [2, 3]. Gastrodin was found to markedly attenuate the expression of the interleukin (IL)1*β*, IL6, and tumor necrosis factor (TNF) both in vivo and in vitro [4]. In vitro and in vivo studies show that the use of FABP4 inhibitors can effectively reduce the inflammatory response, indicating that FABP4 may be a potential candidate target for the treatment of inflammation-related diseases [5–8].

Inflammation is closely related to many metabolic diseases, such as atherosclerosis, type 2 diabetes (T2DM), and obesity [9, 10]. The main immune cell type that causes inflammation in the islets of obesity and type 2 diabetes is macrophages [11]. In addition, macrophages are a major activator of chronic inflammation of the innate immune system and play a central role [12]. FABP4, as a fatty acid companion, is greatly increased when inflammation is activated [13]. In macrophages, the deletion or chemical inhibition of FABP4 will increase the level of intracellular free fatty acids, and the increase of free fatty acids will induce the expression of uncoupling protein 2 (UCP2) through the PPAR*γ*-mediated pathway. The increase in UCP2 expression reduces mitochondrial oxidative stress, endoplasmic reticulum stress, and inflammation and changes the mitochondrial function and the intracellular level of free fatty acids inside macrophages [14–17]. The inflammation endoplasmic reticulum and mitochondrial stress in macrophages can be reduced by inhibiting the binding of FABP4 with free fatty acids in macrophages. Therefore, the development of FABP4 inhibitors can inhibit macrophage inflammation and improve the endoplasmic reticulum and mitochondrial stress of macrophages.

**Figure 1:**
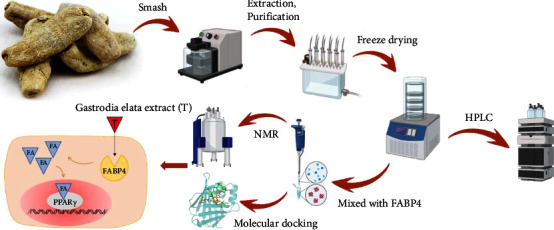
Experimental flowchart.

Based on molecular docking technology, it was found that levofloxacin, a broad-spectrum antibiotic, is also a high-affinity inhibitor of FABP4 [18]. NMR can be used as a method to monitor the intermolecular interaction, and it is a powerful means to study the protein-ligand interaction. Among them, the ligand observation of nuclear magnetic resonance experiments is used to identify small ligand molecules bound to biological macromolecules. Commonly used methods include those based on nuclear Overhauser effect (NOE), saturation transfer difference (STD-NMR), water ligand observed via gradient spectroscopy (WaterLOGSY), and transferred-nuclear Overhauser effect spectroscopy (tr-NOESY) experiments, and also the INPHARMA experiment. The STD-NMR experiment depends on the magnetization exchange from the protein-bound state to the ligand-free state. In this spectrogram, only the signal of the conjugate is observed, but the signal of protein and nonconjugate is subtracted. The conjugate in the mixture can be determined by STD-NMR, and the binding epitope can also be defined [19, 20]. *Gastrodia elata* belongs to Orchidaceae, first recorded in Shennong Materia Medica. As a traditional Chinese herbal medicine, it is generally considered to have anti-inflammatory, antibacterial, and neuroprotective effects. GAS, 4-HBA, etc. are the main components [4, 21]. GAS can increase the activity of superoxide dismutase (SOD) and decrease the level of reactive oxygen species (ROS) in the liver. Some researchers have suggested that the pathway of GAS's efficacy is through the anti-inflammatory effect [22–25]. In this study, GAS, 4-HBA, and phenols with similar structures were isolated from *Gastrodia elata* by column chromatography. After their structures were identified by HPLC and NMR, the 1H-NMR experiment of ligand observation was used to study whether they interacted with FABP4, and the molecular docking technique was used to predict the interaction mode between the active ingredients and proteins with FABP4. The purpose of this study is to find out the active ingredients that inhibit FABP4 from *Gastrodia elata*, which can clarify the molecular mechanism of the anti-inflammatory effect of *Gastrodia elata*, providing the basis for developing the new structure of FABP4 inhibitory drugs (Figure 1).

## 2. Materials and Methods

### 2.1. Materials

Shimadzu LC-16 HPLC is equipped with an SPD-16 UV detector (Shimadzu Company of Japan); a Bruck NMR instrument of 400 MHz (BioSpin Inc.); a KW-1000DC digital display constant temperature water bath (Jiangsu Jintan Yitong Electronics Co., Ltd.); an RE-5229 Rotary evaporator (Zhengzhou Yarong Instrument Co., LTD); an Shz-d (iii) circulating water multipurpose vacuum pump (Zhengzhou Boke Instrument Equipment Co., Ltd.); a BioTek Synergy 2 Multifunctional Enzyme Labeling Instrument (Berten Instrument Co., Ltd., USA); an LGJ-10A freeze dryer (Shanghai Hefan Instrument Co., Ltd.); an FA2004B 1/10,000 electronic analysis balance (Shanghai Yueping Scientific Instrument Co., Ltd.); an FSJ-A03D1 TCM crusher; a KQ3200DB CNC ultrasonic cleaner (Kunshan Testing Instrument Co., Ltd.); and a column chromatography glass column (inner diameter: 2.5 cm).

*Gastrodia elata* is produced in Tongcheng County, Xianning City, Hubei Province. It was identified as *Gastrodia elata* by Associate Professor Xiao Ruolei of the Hubei University of Science and Technology. Standard GAS, 4-HBA, and phenols were purchased from Chengdu Aifa Biotechnology Co., Ltd., with purity >97%; chromatographic grade acetonitrile and methanol were purchased from the United States Symantec Company; HPLC water for Wahaha pure wateer Hangzhou Wahaha Group Co., Ltd. (Hangzhou, China) was used to provide purified water; dichloromethane, trichloromethane, petroleum ether, benzene, ethyl acetate, and ethanol (AR) were purchased from Sinopharm Chemical Reagent Co., Ltd. Dimethyl sulfoxide and Tris-EDTA (pH = 7.5) were purchased from Sigma-Aldrich, USA. FABP4 was purchased from Wuhan Kaisheng Biotechnology Co., Ltd. AB-8, D101, and ADS-7 macroporous resins were purchased from Tianjin Guangfu Fine Chemical Research Institute. Silica gel (200–300 mesh) was purchased from Qingdao Ocean Chemical Co., Ltd.

### 2.2. Extraction and Separation of GAS and 4-HBA

#### 2.2.1. Macroporous Resin Pretreatment

The newly bought macroporous resin was put in 95% ethanol solution overnight, completely immersed in ethanol, and then washed with distilled water and 95% ethanol repeatedly until the filtrate was colorless. The extractant was washed with distilled water until there was no alcohol taste.

#### 2.2.2. Cleaning and Regeneration of Macroporous Resins

The used macroporous resin was soaked with dilute acid and dilute alkali repeatedly, cleaned until the cleaning liquid was colorless, and finally rinsed with distilled water until PH-neutral.

#### 2.2.3. Macroporous Resin Selection

The effects of AB-8, D101, and ADS-7 macroporous resins on GAS and 4-HBA extraction were compared. The dry rhizome of *Gastrodia elata* was sliced and crushed, and 60 g of *Gastrodia elata* powder (3 portions) was taken. 400 mL of 70% ethanol was added for ultrasonic extraction for 1 h. After filtration, the filtrate was concentrated to 30 mL (3 portions) at 70°C and extracted with three kinds of macroporous resin columns (inner diameter: 2.5 cm, 70 g). After gradient elution with water, 30% and 60% ethanol were successively followed by freeze-drying. The separation effect of ab-8 resin with 30% alcohol was the best after HPLC screening.

#### 2.2.4. Selection of Eluent

60 g of rhizoma *Gastrodia elata* powder (3 parts) was weighed and separated with AB-8 resin (detailed steps as shown in 2.2.3). The part eluted with 30% ethanol was evaporated and concentrated, followed by silica gel column chromatography (inner diameter: 2.5 cm, 60 g). Three different eluents were used for separation. The separation efficiency of GAS and 4-HBA by the three eluents was compared. The separated components were concentrated by evaporation, lyophilized, and detected by HPLC.

### 2.3. Extraction of Phenols with Chemical Structure Similar to GAS and 4-HBA

**Figure 2:**
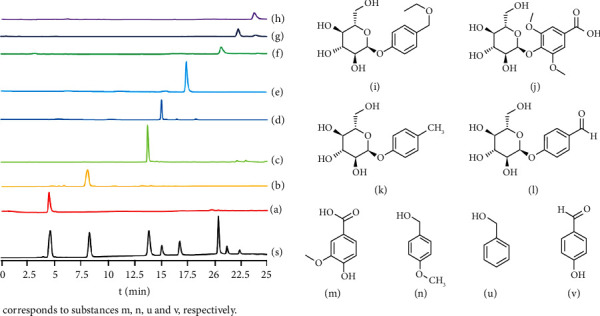
HPLC chromatogram for the extraction of phenols from *Gastrodia elata*. S is the chromatogram of mixed standard, (a)–(d) correspond to substances (i)–(l). (e)–(h) correspond to substances (m)–(v).

*Gastrodia elata* tubers were slice-dried and pulverized. 150 g of Rhizoma *Gastrodia elata* powder was reflux extracted (90% ethanol 500 mL, 2 h × 5) and filtered. The filtrate was concentrated at 70°C into a paste and suspended in water. The mixture was extracted with CH_2_Cl_2_, EtOAc, and n-BuOH to, respectively, obtain fractions soluble in CH_2_Cl_2_ (991.8 mg), fractions soluble in EtOAc (2.43 g), and fractions soluble in n-BuOH (885 mg). The EtOAc fraction was separated by silica gel column chromatography (inner diameter 2.5 cm, 60 g) and eluted by the CHCl_3_-MeOH gradient to obtain four components. Component 2 (0.8 g) was repeatedly separated by petroleum ether-EtOAc to obtain components 1 (12 mg) and 4 (15 mg). Component 3 (0.6 g) was passed through silica gel column chromatography and separated with CHCl_3_-MeOH repeatedly to obtain components 2 (5 mg) and 3 (7 mg). The n-BuOH fraction was subject to silica gel column chromatography (inner diameter 2.5 cm, 50 g) and gradient-eluted with CHCl_3_-MeOH to obtain five fractions, out of which the third fraction (230 mg) was repeatedly separated by benzene-EtOAc to obtain 6 (6 mg) and 7 (9 mg). The CH_2_Cl_2_ fraction was passed through silica gel column chromatography (inner diameter 2.5 cm, 60 g) and eluted with the CHCl_3_-MeOH gradient to obtain four fractions, out of which the second fraction (330 mg) was passed through silica gel column chromatography and repeatedly separated with petroleum ether-EtOAc to obtain components 5 (10 mg) and 8 (8 mg). Each component was dried in a vacuum and identified by HPLC (see Figure 2 for molecular structure).

### 2.4. HPLC Identification Experiment

2 mg of the sample was accurately weighed and dissolved in pure water to a constant volume of 2 mL before being filtered with a 0.45 *µ*m microporous membrane. Standard products are treated with the same method. Chromatography was performed on an Inertsil ODS-3 column (250 nm × 4.6 nm, 5 *µ*m) using two mobile phase conditions: (A) 0.1% phosphoric acid water and (B) acetonitrile. The elution procedure was performed at 0–10 min (for the 6%–15% fraction), at 10–15 min (for the 15%–25% fraction), at 15–25 min (for the 25%–40% fraction), and 25–30 min (for 40% and above). The flow rate was 0.8 mL/min. The column temperature was 30°C. The detection wavelength was 270 nm. The injection volume was 20 *µ*L.

### 2.5. ^1^H-NMR and ^13^C-NMR Identification of GAS and 4-HBA

HPLC can only detect substances in a certain wavelength range. To ensure that fine structure attenuation and chemical shift perturbation can be observed in the ^1^H-NMR spectra of GAS and 4-HBA, ^1^H-NMR and ^13^C-NMR spectra were needed to determine the composition of extracted GAS and 4-HBA, which was convenient for subsequent observation. To prepare for ^1^H-NMR and ^13^C-NMR sample solutions, 5 mg of sample powder was dissolved in 500 *μ*L of DMSO-d_6_.

### 2.6. Ligand Observation by ^1^H-NMR Experiment

50 µg of target protein (FABP4) was dissolved in 2.5 mL of D_2_O and marked as solution A. The extracted GAS and 4-HBA were dissolved in 100 *µ*L of DMSO-d_6_ to make saturated solutions (marked as B and C), and 25 *µ*L of solution B was added to 475 *µ*L of DMSO-d_6_. 25 *µ*L of solution C was added to 475 *µ*L of DMSO-d_6_ to prepare for a protein-free solution, 25 *µ*L of solution B was added to 475 *µ*L of solution A, and 25 *µ*L of solution C was added to 475 *µ*L of solution A to prepare for the protein-containing solution. ^1^H-NMR experiments were performed at 298 K on a Bruker 400 MHz spectrometer, with a relaxation delay of 2 s. The binding phenomenon can be observed by ^1^H-NMR spectrum broadening and chemical shift perturbation.

### 2.7. Molecular Docking Experiment

We generated the structural formula of GAS, 4-HBA, and PPA in Chem Draw 2D 20.0 software (https://revvitysignals.com/products/research/chemdraw). The structure of BMS309403 was downloaded from the PubChem database (https://pubchem.ncbi.nlm.nih.gov/), and we determined the energy optimization of the GAS, 4-HBA, PPA, and BMS309403 structure by using the MM2 force field in Chem Draw 3D 20.0 (https://revvitysignals.com/products/research/chemdraw) [18]. The ligand is saved as a mol2 file after energy optimization. We import the mol2 format of the ligand into Auto Dock Tools 1.5.7 (https://autodocksuite.scripps.edu/adt/) to set the torsion and output it as a pdbqt format file. The crystal structure of FABP4 was downloaded from the protein database RCSB (https://www.rcsb.org) (PDB ID 2NNQ, 1.80 Å). PyMol (https://pymol.org/2/) was then used to remove water molecules and original eutectic ligands from the protein. Proteins were hydrogenated in AutoDock Tools 1.5.7 and output as a file in the pdbqt format. The pdbqt files of the receptor and ligand are imported into Auto Dock Tools 1.5.7. Molecular docking is performed in Auto Dock Tools 1.5.7, and the magnitude of the binding energy reflects the possibility of binding between the receptor and ligand. The lower the binding energy, the higher the affinity between the receptor and the ligand. The lower the binding energy, the more stable the conformation of the receptor and the ligand. In summary, docking was completed in four steps. The first step was protein hydrogenation, charge calculation, and protein type assignment (assign ad4 type). The second step was small molecule hydrogenation to keep it flexible. The third was to set the docking range X88, Y92, and Z116, the docking box completely covers the receptor protein, and the ligand is located outside the docking box. The last step was to use the genetic algorithm for molecular and protein docking using default parameters. After docking for 50 times, visualization was performed in PyMol.

### 2.8. STD-NMR Screening Experiment

50 *µ*g of the target protein (FABP4) was weighed and dissolved in 2.5 mL of NMR buffer solution (10 mM Tris-d_11_ pH 7.8 and 100 mM NaCl) and labeled solution A. The extracted solid powder was dissolved in 100 *µ*L of DMSO-d_6_ to obtain a saturated solution and labeled solution B. The protein-free solution was prepared by adding 25 *µ*L of solution B into 475 *µ*L of NMR buffer solution. The protein-containing solution was prepared by adding 25 *µ*L of solution A into 475 *µ*L of NMR buffer solution. The STD-NMR experiment was achieved by a train of Gauss-shaped pulse stddiffesgp3, in which the water suppression by gradient-tailored excitation (WATERGATE) scheme for the suppression of the residual HDO signal was used. The STD-NMR spectrum of the sample containing the target protein was collected using 32 transients, with a spectral width of 9014.6 Hz, 64 K data points, number of scans at 1024. The STD spectrum was recorded at 25.0 ppm of nonresonant radiation and 0.75 ppm of resonant radiation. All STD effects were quantified using the equation *A*_STD_=(*I*_0_ − *I*_sat_)/*I*_0_=*I*_STD_/*I*_0_. Therefore, signal intensities of the STD-NMR spectrum (*I*_STD_) were compared with the corresponding signal intensities of a reference spectrum (*I*_0_). The strongest STD signal in the spectrum was assigned a value of 100% and used as a reference to calculate the relative STD effects accordingly.

### 2.9. 1,8-ANS Fluorescence Competition Experiment

The fluorescent probe 8-anilinonaphthalene-8-sulfonic acid (1,8-ANS) has been proven to be the ligand of FABP4. When ANS binds to FABP4, its maximum emission light will be blue-shifted. The excitation and emission wavelengths of free ANS were determined to be 370 nm/540 nm in the early stage of the experiment, respectively. After binding protein, it becomes 360 nm/460 nm, so by observing the change in the fluorescence intensity at 360 nm/460 nm before and after adding the ligand, the ability of the ligand to replace bound 1,8-ANS from the binding cavity can be determined.

Competition assay fluorescent experiments: 100 *μ*g FABP4 protein was dissolved in 5 mL Tris-EDTA (pH = 7.4), 10 mg 8-anilino-1-naphthalenesulfonic acid (1,8-ANS) was dissolved in 50 mL distilled water, and 0.5 mL of such 1,8-ANS solution was added to the 4 mL above-mentioned target protein solution; the excitation and emission wavelengths were determined. The GAS solid was added to make the final concentration of GAS (0, 1, 8, 16, 24, 32, 40,4 8, and 56 *μ*M) and mixed in the test tube after adding solid GAS every time; the fluorescence intensity of the solution was measured in a cuvette of Shi Ying using a Hitachi F-7000 fluorescence spectrometer. With the substitution of 1,8-ANS in the FABP4 binding cavity by GAS, the fluorescence intensity gradually decreased, and the fluorescence data of each concentration were recorded. The determination of 4-HBA is the same as above, except that the final concentration of 4-HBA is 0, 10, 20, 30, 40, 50, 60, 70, and 80 *μ*M.

IC_50_ determination experiment: 50 µg of the target protein was dissolved in 2.5 ml of Tris-EDTA (pH = 7.4) buffer solution, 30 mg of ANS was dissolved in 1 mL DMSO to obtain a mother liquor of about 100 mM, 1 *µ*L of the mother liquor was diluted with 2999 *µ*L of Tris-EDTA (pH = 7.4) buffer solution, and 60 *µ*L of diluent was added to a 96-well plate to make the final concentration of ANS 10 *µ*M. Then, 80 *µ*L of the target protein solution was added to a 96-well plate, 60 *µ*L of compound solution obtained by gradient dilution was added as the sample well (the compound was dissolved by Tris-EDTA (pH = 7.4) buffer solution), 60 *µ*L of ANS diluent was added to the 96-well plate, and 80 *µ*L of Tris-EDTA (pH = 7.4) buffer solution was added to the 96-well plate as the local well. 60 *µ*L ANS diluent was added to the 96-well plate to make its final concentration 10 *µ*M; then, 80 *µ*L target protein solution was added to the 96-well plate; compound solution obtained by gradient dilution as blank hole was added; 60 *µ*L ANS diluent was added to the 96-well plate to make its final concentration 10 *µ*M; then, 80 *µ*L Tris-EDTA buffer was added to the 96-well plate; and compound solution obtained by gradient dilution as the control hole was added. The influence of the fluorescence of the compound itself on the results was eliminated. The solution was shaken and incubated at room temperature for 90 s, and then, the fluorescence intensity of each hole was recorded with a BioTek Synergy 2 Multifunctional Enzyme Labeling Instrument for every 45 s at the excitation and emission wavelengths of 360 nm/460 nm for 10 min, and the inhibition rate by the following formula was calculated: Inhibition rate of the sample = (1 − (Fluorescence value of sample hole-Fluorescence value of local hole-Fluorescence value of control hole-Fluorescence value of local hole)/(Fluorescence value of blank hole-Fluorescence value of local hole)) × 100%. The IC_50_ values were expressed as mean from three independent experiments and determined via the nonlinear regression analysis using GraphPad Prism software 9.5.

## 3. Results and Discussion

### 3.1. The Extraction and Separation Results of GAS and 4-HBA

**Figure 3:**
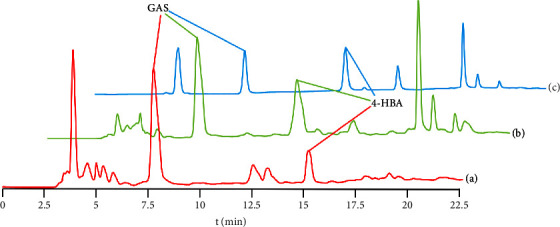
High-performance liquid chromatography of GAS and 4-HBA extracted from three macroporous resins. (a) D101 30% ethanol component, (b) Ab-8 30% ethanol component, and (c) ADS–7 30% ethanol component.

*Gastrodia elata* has a complex chemical composition, so it is difficult to extract and purify. We compared different resins and eluents to optimize the separation condition, obtaining high purity in the final extraction.  Figure 3 is the HPLC characteristic spectrum of the alcohol eluent with the highest content of GAS and 4-HBA among the three resins. The contents of GAS and 4-HBA separated by AB-8 elution are the highest (Figure 3(b)). AB-8 resin is selected for extraction.

**Figure 4:**
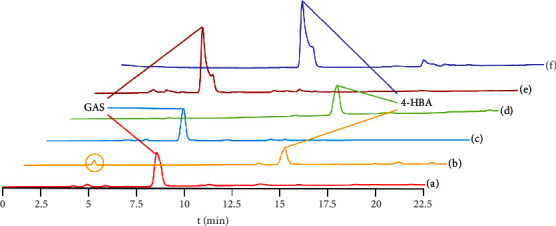
HPLC chromatograms for the separation of GAS and 4-HBA with three eluents. (a) GAS, (b) 4-HBA petroleum ether-EtOAc, (c) GAS, (d) 4-HBA CHCl_3_-MeOH, (e) GAS, and (f) 4-HBA benzene-EtOAc.

Figure 4 shows the HPLC characteristic spectra of different eluents. The separation result of benzene‐EtOAc has an obvious tailing phenomenon, which may be caused by impurities (Figure 4(e) and (f)). The 4-HBA separated by petroleum ether‐EtOAc contains a small amount of impurities (Figure 4(a) and (b)), and the purity of GAS and 4-HBA separated by CHCl_3_‐MeOH as eluent is the highest (Figure 4(c) and (d)), so CHCl_3_‐MeOH is selected.

**Figure 5:**
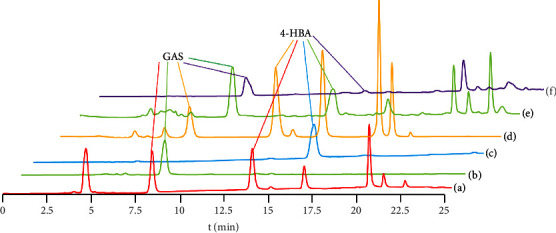
HPLC chromatogram of GAS and 4-HBA extraction results. Standard (a) GAS 8.340 min; 4-the HBA 13.937 min. 100 : 40 chloroform-methanol extract (b) 8.368 min. 100 : 12 chloroform-methanol extract (c) 13.928 min. Ab-8 macroporous resin eluent water (d), 30% (e) ethanol, and 60% ethanol (f).

**Figure 6:**
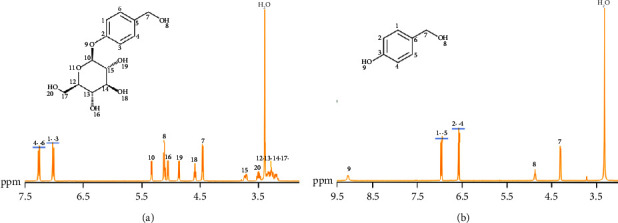
^1^H‐NMR of GAS and 4-HBA. The numbers in the ^1^H-NMR spectrum represent the hydrogen atoms at this position in the molecular formula. (a) GAS ^1^H-NMR and (b) 4-HBA ^1^H‐NMR.

Figure 5 is the HPLC characteristic spectra of the extraction and separation results of GAS and 4-HBA. R4, R5, and R6 are HPLC chromatograms of macroporous resin AB-8 eluted with water, 30% ethanol, and 60% ethanol, respectively. It is found that the chromatographic signals of GAS and 4-HBA in 30% ethanol eluate Figure 5(e) are relatively strong, so 30% components are selected to be separated.  Figure 6(a) shows that the retention time of standard GAS is 8.340 min and that of 4-HBA is 13.937 min; R3 is 100 ∶ 12. The retention time of the CHCl_3_‐MeOH extract is 13.928 min (Figure 5(b)); R3 is 100 ∶ 40. The retention time of CHCl_3_‐MeOH extract is 8.368 min. Therefore, Figure 5(b) was determined as GAS; and Figure 5(c) was determined as 4-HBA.

### 3.2. Analysis of Phenolic Extraction Results of *Gastrodia elata*

The structure of phenols was identified by high-efficiency liquid chromatography. A comparison of chromatogram of mixed calibrating standards and phenolic extract (Figure 2(s)). The reference substance found that Figure 2(a)–(d) in HPLC diagram corresponds to substances i, j, k, and l, respectively; Figure 2(e)–(h) corresponds to substances m, n, u, and v, respectively.

### 3.3. Analysis of ^1^H-NMR and ^13^C-NMR Experimental Results

**Figure 7:**
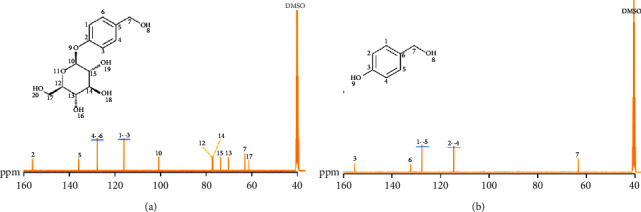
^13^C‐NMR of GAS and 4-HBA. The number in the ^13^C-NMR spectrum indicates the carbon atom at this position in the molecular formula. (a) GAS ^13^C‐NMR and (b) 4-HBA ^13^C‐NMR.

GAS ^1^H-NMR showed two groups of aromatic hydrogen proton signals: *δ*: 7.22 (d, *J* = 8.6 Hz, 2H) and 6.97 (d, *J* = 8.6 Hz, 2H) (Figure 6(a)); the carbon spectrum gives an aromatic carbon signal in low field regions 156.11, 135.64, 127.50, and 115.7 3 (Figure 7(a)), which indicates that 1,4-disubstituted benzene exists in the molecule; C-terminal hydroxyl signal of sugar 4.82 (d, J = 7.3 Hz, 1H) (Figure 6(a)), carbon spectrum shows the carbon signal of sugar 100.34, 76. 80, 76.44, 73.05, 69.54, and 62.31 (Figure 7(a)), consistent with glucopyranose. GAS ^1^H-NMR (400 MHz, DMSO-d_6_) *δ*: 7.22 (*d*, *J* = 8.6 Hz, 2H), 6.97 (*d*, *J* = 8.6 Hz, 2H), 5.29 (*d*, *J* = 4.8 Hz, 1H), 5.07 (*t*, *J* = 5.5 Hz, 1H), 5.01 (*d*, *J* = 5.3 Hz, 1H), 4.82 (*d*, *J* = 7.3 Hz, 1H), 4.55 (*t*, *J* = 5.8 Hz, 1H), 4.42 (*d*, *J* = 5.6 Hz, 2H), 3.13–3.75 (6H, m, 12, 13, 14, 15, 17, 20-H) identified as GAS (Figure 6(a)). It is consistent with references [26, 27].

4-HBA ^1^H-NMR showed two groups of aromatic hydrogen proton signals, *δ*: 7.09 (*d*, *J* = 8.4 Hz, 2H) and 6.69 (*d*, *J* = 8.5 Hz, 2H) (Figure 6(b)), and a hypomethyl proton signal 4.35 (*d*, *J* = 4.6 Hz, 2H). Combined with carbon spectrum data, it was identified as 4-HBA (Figure 7(b)). Hydrogen spectrum data of 4-HBA, 1H-NMR (400 MHz, DMSO-d6) *δ* 9.24 (s, 1H), 7.09 (*d*, *J* = 8.4 Hz, 2H), 6.69 (*d*, *J* = 8.5 Hz, 2H), 4.94 (*t*, *J* = 5.7 Hz, 1H), 4.35 (*d*, *J* = 4.6 Hz, 2H) identified as 4-HBA (Figure 6(b)). It is consistent with references.

Figure 7(a) shows the extracted GAS: ^13^C-NMR (DMSO-d_6_, 400 MHz) *δ*: 156.11 (C-2), 135.64 (C-5), 127.50 (C-4, 6), 115.73 (C-1, 3), 100.34 (C-10), 76. 80 (C-12), 76.44 (C-14), 73.05 (C-15), 69.54 (C-13), 62.31 (C-7), and 60.52 (C-16). Figure 7(b) shows the extracted 4-HBA: ^13^C-NMR (DMSO-d_6_, 400 MHz) *δ*: 156.17 (C-2), 132.74 (C-5), 128.04 (C-4, C-6), 114.77 (C-1, C-3), and 62.77 (C-7). It is consistent with references [26, 27].

### 3.4. ^1^H-NMR Experimental Results of Ligand Observation

As a method to monitor the intermolecular interaction, NMR is a powerful means to study the protein-ligand interaction. Among them, the NMR experiments of ligand observation can be used to identify small ligand molecules combined with biological macromolecules [28]. NMR observations such as transverse and longitudinal relaxation rates (*R*1 and *R*2), nuclear Overhauser effect (NOE), and diffusion coefficient are highly dependent on the molecular size and shape. Therefore, the NMR parameters of small molecules are very sensitive to their interactions with large molecules. If a small molecule binds to a protein, the chemical environment of the hydrogen nucleus at this site will change, which will disturb the chemical shift of this site. The changes in the chemical shift, line width, relaxation rate, and NOE value can be used to characterize and quantify the binding of small molecules to larger target molecules such as proteins. Macromolecules show smaller diffusion coefficients and larger *R*2 values, and there is a more obvious NOE effect between nuclei, while small molecules are the opposite. When a small molecule binds to a protein, it will temporarily appear as the NMR observation of the protein. Due to the large longitudinal relaxation rate (*R*2) of the protein and the more obvious NOE effect between nuclei, small molecules bound to the protein will show wider spectral lines and attenuation of fine structures. Through these and chemical shift perturbation, the ligand small molecules bound to protein can be selected.

**Figure 8:**
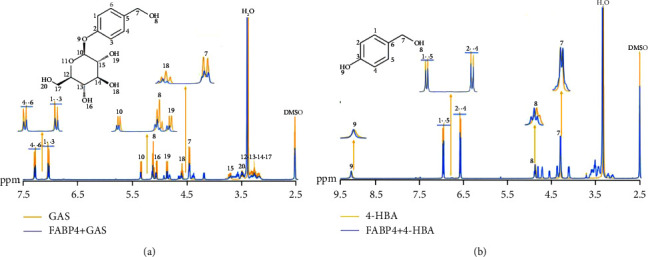
^13^H-NMR experiment for ligand observation. The numbers in the ^1^H-NMR spectrum represent the hydrogen atoms at this position in the molecular formula. (a) GAS ^1^H-NMR experiment for ligand observation and (b) 4-HBA ^1^H-NMR experiment for ligand observation.

100 ∶ 12 and 100 ∶ 40 CHCl_3_-MeOH extracts were identified as GAS and 4-HBA by HPLC and NMR. GAS and 4-HBA combined with FABP4 protein will temporarily show the NMR observation values of protein molecules, so we can compare the changes in ^1^H-NMR spectra of GAS and 4-HBA before and after protein addition. The attenuation of fine structure, broadening of spectral line, and perturbation of chemical shift, and whether GAS and 4-HBA are bound to protein can be judged from Figure 8(a), which shows the ^1^H-NMR spectrum of GAS before and after protein addition. It is observed that the fine structure of all hydrogen nuclei has been attenuated, and the chemical shift perturbation of No. 8, No. 10, No. 19, and No. 20 hydrogen atoms has been observed (their chemical shifts move to the low field, possibly due to the formation of hydrogen bonds between No. 8, No. 10, No. 19, and No. 20 hydrogen atoms and proteins, leading to the decrease in the electron cloud density of this hydrogen atom, so the chemical shift of hydrogen nuclei moves to the low field). The above indicates that there is a binding phenomenon between GAS and protein.  Figure 8(b) shows the ^1^H-NMR spectrum of 4-HBA before and after protein addition. We observed the attenuation of the fine structure of No. 1, No. 3, No. 4, and No. 6 hydrogen atoms, as well as the perturbation of chemical shifts of hydroxyl hydrogen atoms 8 and 9, and the broadening of the spectral line of hydrogen atom 7. Therefore, GAS and 4-HBA are binding with protein.

### 3.5. Analysis of Docking Results of GAS and 4-HBA Molecules

**Figure 9:**
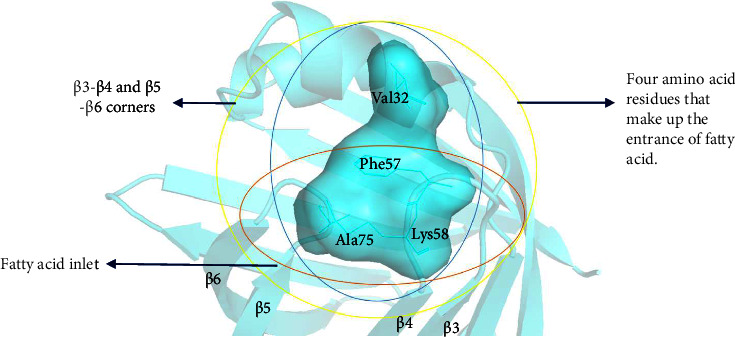
FABP4 protein entrance position composition.

FABP4 shows a typical *β*-barrel structure, and the cavity in the middle is the binding site of ligands such as fatty acids. According to the ligand entrance hypothesis of FABP4, the fatty acid entrance consists of the angle between *β*3-*β*4 and *β*5-*β*6 and the amino acid residues of *α* helix Val32, Phe57, Lys58, and Ala75 (Figure 9). The entrance position change allows the ligand to enter mainly based on the conformational change mechanism of Phe57. The carboxyl group of FAs is oriented inward and coordinates with one tyrosine and two arginine residues through electrostatic interaction (the bonded fatty acid carboxyl group forms a salt bridge with Arg106, Arg126, and Tyr128, and the mutation of each of these amino acids will cause the protein to be unable to bond with fatty acids) [29].

**Figure 10:**
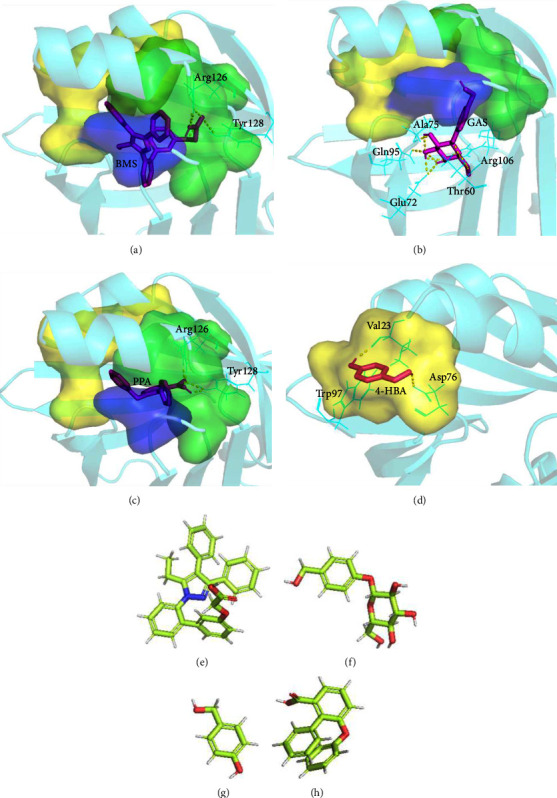
Molecular docking results. (a) BMS309403 and FABP4, (b) GAS and FABP4, (c) PPA and FABP4, (d) 4-HBA and FABP4, the yellow area is the amino acid residue of 4 Å near 4-HBA. (e) BMS309403, (f) GAS, (g) 4-HBA, and (h) PPA chemical structure. Hydrophobic regions 1 and 2 in (a), (b), and (c) are consistent with Figure 11. Pink represents the small molecules, yellow spheres represent the hydrogen bonds, and blue molecules represent the amino acid residues.

**Figure 11:**
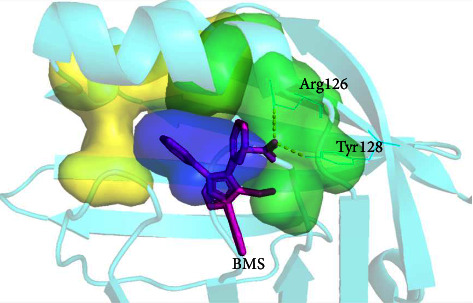
The eutectic structure of BMS and FABP4 protein (PDB ID:2NNQ). Yellow (Phe16, Tyr19, Met20, Val25, and Asp76) and blue-purple (Phe57) form hydrophobic region 1. Green (Ala33, Gly34, Ala36, Pro38, Ser55, and Arg126) and blue-purple (Phe57) form hydrophobic region 2.

The molecular docking results are shown in (Figure 10). The binding site of BMS309403 and FABP4 is at the fatty acid inlet (Figure 10(a), Table S1). The carboxyl group of BMS309403 forms hydrogen bonds with Arg126 and Tyr128, and the total binding energy is −10.95 kcal/mol. The aromatic group at position 3 of the central nucleus of BMS309403 pyrazole is in the hydrophobic region 1 formed by Phe16, Tyr19, Met20, Val25, Phe57, and Asp76 (hydrophobic region 1 composed of yellow and purple Phe57 amino acid residues in Figure 10(a)). The aromatic ring at position 4 is located in hydrophobic region 2 formed by Ala33, Gly34, Ala36, Pro38, Ser55, Phe57, and Arg126 (hydrophobic region 2 composed of green and blue-violet Phe57 amino acid residues in Figure 10(a)). The eutectic structures of BMS309403 and FABP4 (PDB ID: 2NNQ (Figure 11) are consistent with molecular docking results [30, 31].

The docking results of FABP4 and GAS (Figure 10(b), Table S2) show that hydrophilic glucose forms hydrogen bonds with Arg106, Ala75, Gln95, Glu72, and Thr60, and the total binding energy is −6.45 kcal/mol. The benzene ring of GAS is located in hydrophobic region 2, which is consistent with the position of the 4-position benzene ring of BMS309403 in protein. Although the number of hydrogen bonds formed between GAS and protein is more than BMS309403, the total bond energy is lower than the latter, which may be due to the hydrophobic interaction between BMS309403 and hydrophobic regions. It can be seen that the hydrophobic interaction formed by the two hydrophobic regions is one of the key acting forces for the combination of BMS309403 and FABP4.

The docking result of FABP4 and 4-HBA is shown in Figure 10(d) and Table S3; two hydroxyl groups of 4-HBA form hydrogen bonds with protein amino acid residues Trp97, VAL23, and Asp76 to obtain a total binding energy of −4.2 kcal/mol. The binding position is at the entrance of protein and fatty acid binding. The docking results of FABP4 and PPA (Figure 10(c), Table S4) show that carboxyl groups of PPA form hydrogen bonds with Arg126 and Try128, and the total binding energy is −7.81 kcal/mol, which is higher than −4.2 kcal/mol. However, the number of hydrogen bonds is less than that of GAS, and the two benzene rings are in hydrophobic regions 1 and 2, which shows that the binding mode is similar to BMS309403. The binding sites of BMS309403, GAS, 4-HBA, and PPA are all at the entrance where fatty acids bind to FABP4 protein.

The docking results indicate that GAS, 4-HBA, and PPA may exert similar inhibitory effects as BMS309403 and, thus, exert similar pharmacological activities. GAS, 4-HBA, and phenols with similar structures are all active ingredients in *Gastrodia elata*. Therefore, after taking *Gastrodia elata*, they may play a synergistic role in inhibiting FABP4, and their binding energy is lower than that of BMS309403. Therefore, compared with BMS309403, the effect of the *Gastrodia elata* active ingredient is mild.

### 3.6. Experimental Results of STD-NMR

STD-NMR experiment depends on the magnetization exchange from the protein-bound state to the ligand-free state. The method includes selectively saturating protein NMR signals by using certain radiofrequency radiation without interfering with the resonance signal of small molecules. After saturation, it passes through NOE and spin diffusion. Therefore, all protein resonances are saturated. When the molecule binds to the protein surface, the saturation of the protein proton is transferred to the molecule through the spin diffusion of intermolecular NOE, which leads to the proton saturation of the small molecule bound to the protein. As the protein and ligand are constantly bound and dissociated, the small molecule is constantly saturated, but the molecule that is not bound to the protein will not change. To attenuate that resonance signal of the small molecule bound to the saturated protein, and through protein resonance unsaturation (the protein and the ligand are not resonantly saturated so that the nuclear magnetic spectrum shows the signals of all proteins and ligand) and resonance saturation (the protein is resonantly saturated under a certain radio frequency signal so that the ligand signal bound to the protein is also saturated), STD-NMR can be used to determine the conjugate in the mixture, and the binding epitope (the part that binds to the protein most closely) can also be defined.

Because STD-NMR only shows the proton signal of the ligand bound to protein, the bound ligand can be visually observed from the STD-NMR spectrogram, and the 1.8-ANS fluorescence competition experiment is used to further verify the small molecule with strong signal.

**Figure 12:**
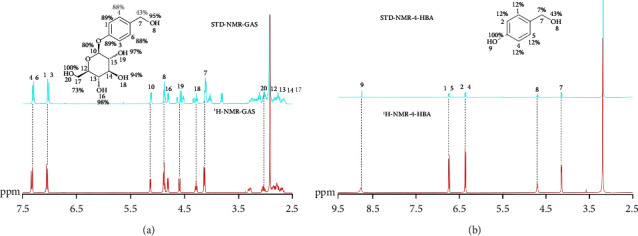
STD-NMR results. STD (blue) and reference ^1^H-NMR (red) NMR spectra acquired. (a) GAS and FABP4, (b) 4-HBA and FABP4.

The results of STD-NMR showed that the hydrogen atoms of GAS and 4-HBA interacted with FABP4, and the STD-NMR signal of GAS was strong, while that of 4-HBA was weak, which indicated that the interaction between gas and FABP4 was stronger than that of 4-HBA, which is consistent with the molecular docking results (Figures 12(a) and 12(b)). In the STD-NMR spectrum of 4-HBA, the signals of No. 8 and No. 9 hydrogen atoms are stronger than those of other hydrogen atoms, indicating that these two hydrogen atoms have a stronger interaction with FABP4, which is consistent with the docking result that these two hydrogen atoms form hydrogen bonds with FABP4 (Figure 12(b)). In the STD-NMR spectrum of GAS, it is found that hydroxyl groups mainly interact with FABP4, and the proportion of hydrogen atoms in the benzene ring is also high, indicating that there may be *π*-*π* interaction between GAS and amino acid residues of FABP4(Figure 12(a)). All STD effects were quantified using the equation *A*_STD_=(*I*_0_ − *I*_sat_)/*I*_0_=*I*_STD_/*I*_0_. Therefore, signal intensities of the STD-NMR spectrum (*I*_STD_) were compared with the corresponding signal intensities of the reference spectrum (*I*_0_). The strongest STD signal in the spectrum was assigned a value of 100% and used as the reference to calculate the relative STD effects accordingly.

### 3.7. 1.8-ANS Fluorescence Competition Experiment Results

1.8-ANS is proved to be the ligand of FABP4, which can lead to an increase in the fluorescence intensity and the blue shift of the maximum emission light after binding with protein. Before the experiment, the excitation wavelength and emission wavelength of free 1.8-ANS were determined by spectral scanning, and after binding to the protein, the excitation wavelength and emission wavelength of the increased fluorescence intensity were determined. The addition of inhibitors can compete with 1.8-ANS for binding sites, resulting in 1.8-ANS in its free form, leading to a decrease in the fluorescence intensity. Increasing the concentration of inhibitors would increase free-form 1.8-ANS, leading to a decrease in the fluorescence intensity. The fluorescence intensity will not decrease if it is not bound to protein, so it can be used for the simple screening of small molecule inhibitors bound to protein [32–35].

**Figure 13:**
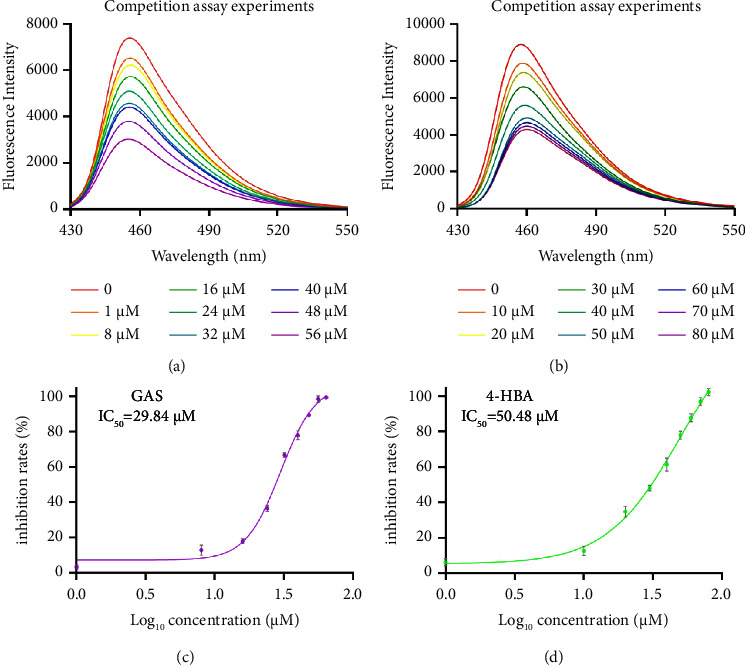
Fluorescence competition experiment and half inhibitory concentration (IC_50_) results. (a) GAS and (b) 4-HBA fluorescence competition experiment results. (c) GAS and (d) 4-HBA half inhibitory concentration (IC_50_) results.

The results of fluorescence competition experiments show that the fluorescence intensity decreases with the addition of different concentrations of GAS and 4-HBA compared with that without GAS and 4-HB, indicating that GAS and 4-HBA compete with 1,8-ANS for binding sites, which leads to a decrease in the concentration of ANS-FABP4 in the system and a decrease in the fluorescence intensity (Figure 13(a) GAS, Figure 13(b) 4-HBA). The IC_50_ of GAS and 4-HBA were calculated to be 29.84 *μ*M and 50.48 *μ*M (Figure 13(c) d), respectively, which is consistent with the results of molecular docking and STD-NMR, and both of them competed with ANS for binding sites, indicating that their binding sites were in the binding cavity of FAPB4 and ligand, and ANS bound to FAPB4 could be replaced.

## 4. Conclusion

Different macroporous resins and silica gel column chromatography eluents were compared. AB-8 macroporous resin and silica gel (200–300 mesh) were selected as the stationary phase, and CHCl_3_-MeOH was used as the eluent of the silica gel column. Eight phenolic monomers, such as GAS and 4-HBA (the main active components extracted from *Gastrodia elata*), are adopted by column chromatography, and the content and purity are high (95%). Their structures were identified by HPLC and NMR, and it was found that their structures were similar to those of GAS and 4-HBA. By observing the attenuation of the peak intensity and the perturbation of chemical shift in the ^1^H-NMR of the ligand with or without protein, it was found that the binding phenomenon occurred among GAS, 4-HBA, and FABP4. The molecular docking technique was used to predict the space position of their binding with FABP4. By using the 1,8-ANS fluorescence competition experiment, it was found that the binding site of GAS and 4-HBA was in the binding cavity of FABP4 and ligand. The results showed that they were similar to the classical FABP4 inhibitor BMS309403, but their binding energy was low. The IC_50_ concentrations of GAS and 4-HBA were 29.84 *μ*M and 50.48 *μ*M. However, a variety of phenols with similar structures to GAS and 4-HBA were found in *Gastrodia elata*, which may be the common inhibition of FABP4 by a variety of components with similar structures. Meanwhile, it also provides a new strategy to inhibit FABP4, i.e., a variety of substances with similar structures but mild effects play an inhibitory role together, to play a role in resisting macrophage inflammation. At the same time, it is also helpful to the research of other metabolic diseases. At last, PPA was found to be a possible structure of the FABP4 inhibitor by molecular docking technology, providing new strategies and useful data for the drug research of the FABP4 inhibitor, creating a new basis for the molecular mechanism of anti-inflammation of *Gastrodia elata* [36–38].

## Data Availability

The data used to support the findings of this study are available from the corresponding author upon request.

## Supplementary Materials

Supplementary Materials Table S1–S4: cluster analysis of molecular docking results.
