# Supplementary material for: The Molecular Mechanism of FABP4 Inhibition Effects of GAS and 4-HBA in Gastrodia elata Blume Was Discussed Based on NMR and Molecular Docking
Source: J Anal Methods Chem. 2024 May 8;2024:6599029. doi: 10.1155/2024/6599029 (PMC11095988; doi:10.1155/2024/6599029)
Supplement: Supplementary Materials — Table S1–S4: cluster analysis of molecular docking results. [file 6599029.f1.docx]

**The Molecular Mechanism of FABP4 Inhibition Effects of GAS and 4-HBA in *Gastrodia elata* Blume was Discussed Based on NMR and Molecular Docking**

Yuyu Yang^a^, Shihan Liu^a^, Wenfang Jin^a^, Zengyi Qu^a^, Baolei Fan^a^

^a^Hubei University of Science and Technology, No.88, Xianning Avenue, XiananDistrict, Xianning 437000, China

*Corresponding author: Baolei Fan

Baolei Fan: [fanbl_1980@163.com](mailto:fanbl_1980@163.com)

1.Cluster analysis of molecular docking results

Table S1. Cluster Analysis of Molecular Docking between BMS309403 and FABP4

| Cluster Rank | Lowest Binding Engergy | Run | Mean Binding Energy | Nnm in Clus |
| --- | --- | --- | --- | --- |
| 1 | -10.95 | 49 | -10.24 | 8 |
| 2 | -10.76 | 21 | -9.92 | 10 |
| 3 | -10.68 | 36 | -9.94 | 14 |
| 4 | -10.53 | 2 | -10.20 | 4 |
| 5 | -10.27 | 14 | -9.27 | 4 |
| 6 | -9.82 | 5 | -9.52 | 2 |
| 7 | -9.60 | 38 | -9.60 | 1 |
| 8 | -9.59 | 27 | -9.59 | 1 |
| 9 | -9.43 | 39 | -9.08 | 2 |
| 10 | -9.15 | 31 | -9.15 | 1 |
| 11 | -8.82 | 1 | -8.82 | 1 |
| 12 | -8.37 | 45 | -8.37 | 1 |
| 13 | -8.34 | 46 | -8.34 | 1 |

Table S2. Cluster Analysis of Molecular Docking between GAS and FABP4

| Cluster Rank | Lowest Binding Engergy | Run | Mean Binding Energy | Nnm in Clus |
| --- | --- | --- | --- | --- |
| 1 | -6.45 | 41 | -5.84 | 15 |
| 2 | -5.86 | 29 | -5.10 | 3 |
| 3 | -5.79 | 35 | -5.08 | 14 |
| 4 | -5.03 | 8 | -4.69 | 4 |
| 5 | -4.87 | 45 | -4.87 | 1 |
| 6 | -4.83 | 19 | -4.83 | 1 |
| 7 | -4.79 | 15 | -4.79 | 1 |
| 8 | -4.73 | 50 | -4.73 | 1 |
| 9 | --4.58 | 9 | -4.28 | 3 |
| 10 | -4.53 | 5 | -4.53 | 1 |
| 11 | -4.48 | 4 | -4.48 | 2 |
| 12 | -4.46 | 30 | -4.46 | 1 |
| 13 | -3.91 | 44 | -3.91 | 1 |
| 14 | -3.83 | 27 | -3.83 | 1 |
| 15 | -3.82 | 21 | -3.82 | 1 |

Table S3 Cluster Analysis of Molecular Docking between 4-HBA and FABP4

| Cluster Rank | Lowest Binding Engergy | Run | Mean Binding Energy | Nnm in Clus |
| --- | --- | --- | --- | --- |
| 1 | -4.20 | 24 | -4.14 | 17 |
| 2 | -4.10 | 1 | -4.08 | 13 |
| 3 | -4.08 | 49 | -4.01 | 2 |
| 4 | -4.07 | 2 | -4.07 | 1 |
| 5 | -3.99 | 39 | -3.98 | 7 |
| 6 | -3.97 | 11 | -3.97 | 1 |
| 7 | -3.92 | 44 | -3.91 | 3 |
| 8 | -3.89 | 47 | -3.89 | 1 |
| 9 | -3.78 | 25 | -3.70 | 4 |
| 10 | -3.69 | 8 | -3.69 | 1 |

Table S4. Cluster Analysis of Molecular Docking between PPA and FABP4

| Cluster Rank | Lowest Binding Engergy | Run | Mean Binding Energy | Nnm in Clus |
| --- | --- | --- | --- | --- |
| 1 | -7.81 | 2 | -7.56 | 44 |
| 2 | -7.29 | 8 | -7.26 | 3 |
| 3 | -6.56 | 32 | -6.56 | 1 |
| 4 | -5.67 | 14 | -5.67 | 1 |
| 5 | -4.81 | 38 | -4.81 | 1 |
